# Fully robotic extended total gastrectomy with transthoracic long Roux limb reconstruction

**DOI:** 10.1007/s00464-026-12745-1

**Published:** 2026-03-18

**Authors:** Adam Zeyara, Marc J. van Det, Franco Badaloni, Jelle P. Ruurda, Henk-Jan T. J. Mantel, Ewout A. Kouwenhoven

**Affiliations:** 1 Department of Surgery, ZGT Almelo, Almelo, The Netherlands; 2 Department of Surgery, UMC Utrecht, Utrecht, The Netherlands; 3 Department of Surgery, Skane University Hospital, Lund, Sweden

**Keywords:** Robotic, Extended total gastrectomy, Long Roux limb

## Abstract

**Background/Aim:**

Achieving negative margins while ensuring a safe reconstruction is known to be challenging in junctional tumors involving both the stomach and the distal esophagus. When a gastric conduit or a transhiatal Roux limb is precluded, a transthoracic long Roux limb reconstruction offers an alternative. Open approaches have traditionally carried high morbidity rates, but advances in minimally invasive surgery could possibly mitigate these limitations. The primary aim of this study was to assess the feasibility and safety of a fully robotic approach in a highly selected patient population treated in a specialized surgical setting.

**Methods:**

All fully robotic extended total gastrectomies with transthoracic long Roux limb reconstruction performed at the ZGT Hospital in Almelo, The Netherlands, were extracted from a prospective registry. Data were summarized in tables. Means and medians were calculated as appropriate. Survival was calculated by means of Kaplan–Meier analyses.

**Results:**

Twenty-three patients were included, most with cT3–4N + tumors and unfavorable histology. Major complications (Clavien–Dindo ≥ 3) occurred in five (22%), and anastomotic leaks in two (8.7%) patients. Thirty- and 90-day mortality rates were 0% and 9%, respectively. Mean operative time was 7 h, with median ICU and hospital stays of 1 and 9 days, respectively. The mean lymph node yield was 26.5. There were five (22%) R1 resections, all of which had diffuse histology and intraoperative endoscopic as well as pathological assessment. The median overall and disease-free survival was 13.1 and 6.4 months, respectively.

**Conclusion:**

Outcomes indicate that the technique is feasible and safe, possibly offering favorable recovery compared to traditional open methods. Oncologic outcomes are reported descriptively and should be interpreted in the context of advanced disease stage and unfavorable tumor biology. A relatively high rate of R1 resections was observed, highlighting the challenges in the represented cancer subtypes rather than procedural deficits.

**Supplementary Information:**

The online version contains supplementary material available at 10.1007/s00464-026-12745-1.

Achieving negative surgical margins while ensuring a safe reconstruction is known to be challenging in the management of junctional tumors involving both the stomach and the distal esophagus [1, 2]. When the use of a gastric conduit or transhiatal Roux limb is precluded, alternative types of reconstruction must be employed. In addition to the technical challenges, these tumors frequently represent aggressive adenocarcinoma subtypes (diffuse, poorly cohesive, signet cell), further complicating surgical management from a biological perspective. Contributing factors include commonly advanced stage at presentation, inability of an accurate endoscopic assessment of tumor borders, macroscopically diffuse tumor borders, and low specificity of negative frozen sections.

Reconstructive options in these cases include a long Roux limb or colonic interposition [3, 4]. The long Roux limb is generally considered a safe and reliable reconstructive option, in terms of both long-term outcomes and quality of life. However, its achievable length is somewhat unpredictable, varying between patients and limited by the number of jejunal arteries that can be safely divided. For pedicled jejunal grafts, viability is often compromised when more than two or three jejunal arteries are divided [1, 5]. If a Roux limb is the only reconstructive option and a very proximal anastomosis is warranted, a vascularly augmented, so-called supercharged, jejunal graft can be employed [6–8]. As to colonic interpositions, length is seldom an issue, but specific preparations are needed (preoperative colonic enema), the surgical procedure is more complex, and from a functional perspective, they are prone to senescent lengthening and often subjected to revisional surgery [3, 9–12].

Reconstruction using a long Roux limb is usually performed with a transthoracic (two-field) approach, either as separate incisions or a so-called thoraco-phrenico-abdominal approach [1]. The transhiatal approach has traditionally been limited by anatomic conditions and how big you can make a phrenotomy for access to the mediastinum (in open surgery) as well as non-flexible instruments (in laparoscopy). Nowadays, with the articulating arms of the robot, even though somewhat more accessible, there is still a clear limitation of how proximal you can go. This is where the transthoracic long Roux limb reconstruction may offer some advantages (pedicled jejunal grafts can even reach as high as the azygos vein) and the possibility to perform a complete thoracic D2 lymphadenectomy (which may be necessary as locally advanced junctional cancers do not always conform neatly to the Siewert classification) [13]. Open transthoracic approaches carry high morbidity rates. However, recent advances in minimally invasive techniques in esophagogastric surgery could potentially mitigate the drawbacks of this approach. In our center, we have performed fully robotic extended gastrectomies with transthoracic long Roux limb reconstruction since 2016. The aim of this study was to assess feasibility and safety as well as oncologic and survival outcomes of this fully robotic approach. To the best of our knowledge, this represents the first-reported series describing a fully robotic transthoracic long Roux limb reconstruction following extended total gastrectomy.

## Methods

### Data collection

Granular data on all fully robot-assisted extended total gastrectomies with a transthoracic long Roux limb reconstruction performed at the ZGT Hospital in Almelo, the Netherlands, in the time frame 2016–01–01 (inception)–2024–12–31 were collected from a prospective registry database and validated/supplemented from electronic medical records.

An extended Roux limb was defined as a jejunal Roux limb mobilized by selective division of proximal jejunal mesenteric vessels, allowing a tension-free esophagojejunostomy with increased effective limb reach compared with standard Roux-en-Y reconstruction.

Anastomotic leak was defined according to the ECCG criteria.

Pneumonia was defined as a new infiltrate on chest radiograph associated with clinical signs of infection.

### Statistics

All data were summarized in tables and categorized for patient, preoperative tumor characteristics, operative, postoperative, and pathology data.

Continuous values were presented as mean (± SD) or median (IQR) depending on their normality of distribution.

Kaplan–Meier analysis was used to evaluate survival outcomes.

All statistical analyses were performed using the IBM SPSS Statistics 30.0 (Build 172) software for Mac.

All statistical figures were created using Matplotlib for Python.

### Outcomes

The aim of this study was to assess the feasibility and safety as well as oncologic and survival outcomes of this fully robotic approach.

### A summarized point-by-point description of the procedure

**Fig. 1:**
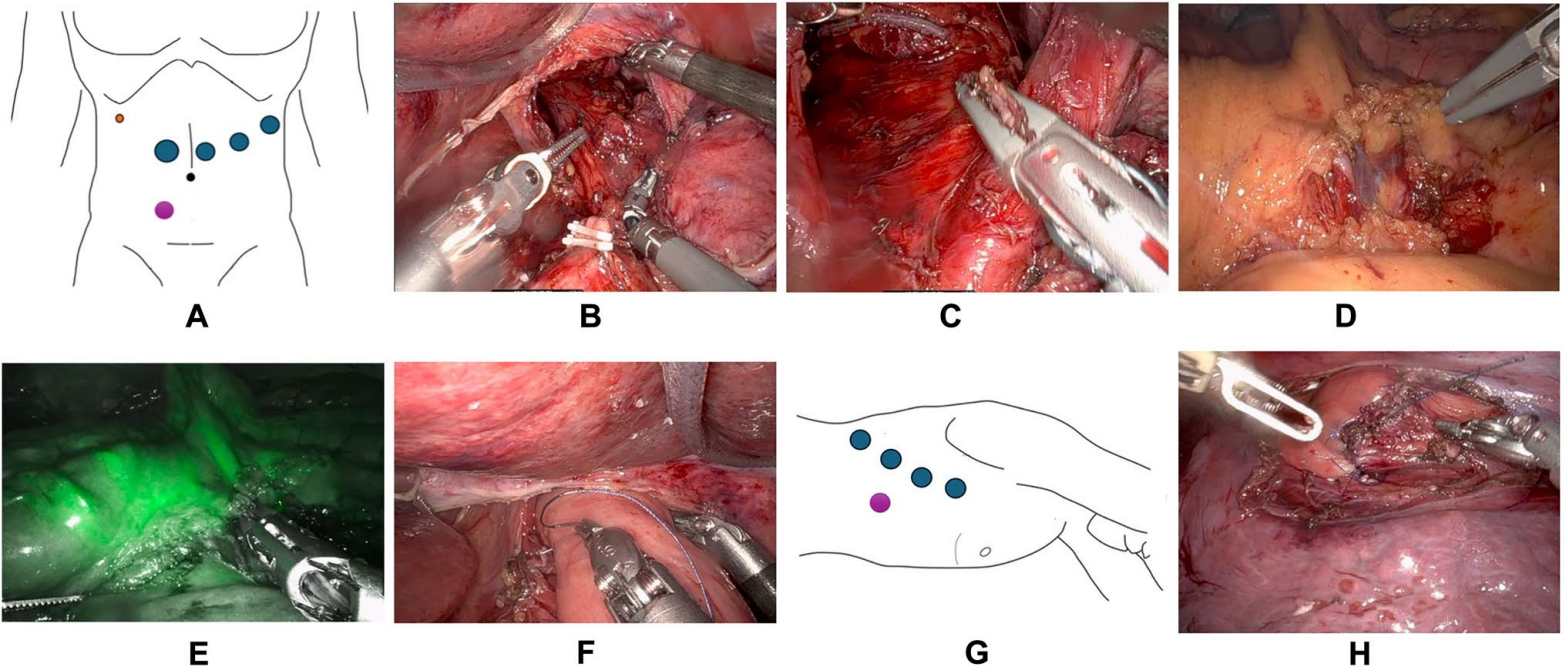
**A** Trocar positions for abdominal phase, **B** Circumferent esophageal mobilization high up in the mediastinum, **C** Transection of the esophagus high up in the mediastinum, **D** Isolation of jejunal vessels of the long roux limb, **E** Before division of jejunal vessels an ICG-control is performed with the vessels clamped to ensure adequate perfusion, **F** The long roux limb is brought up to the right pleura and sutured to the right crus, **G** Trocar positions for thoracic phase, **H** An end-to-side esophagojejunostomy is performed transthoracically

All procedures were performed using the DaVinci Xi platform (Intuitive Surgical Inc) (Fig. 1).

#### Abdominal phase

The patient is positioned supine in the French position and tilted in slight anti-Trendelenburg (15–20°). Four robotic trocars (three 8 mm and one 12 mm) are inserted. Additional incisions include a 5-mm subcostal incision for the REVEEL^®^ liver retractor (Microline Surgical, Inc., Beverly, MA, USA) and an 8 mm assistant port incision. See Fig. 1A for trocar positions. The dissection part of the abdominal phase is performed according to the standard technique for robot-assisted minimally invasive gastrectomy (RAMIG) [14]. The esophagus is then mobilized as high into the mediastinum as possible (Fig. 1B) and transected using the SureForm™ robotic stapler (Intuitive Surgical, Inc., Sunnyville, CA, USA) with a blue reload cartridge, either during this phase or later in the thoracic phase (Fig. 1C). A long Roux limb is created intracorporeally. Jejunal vessels arising directly from the superior mesenteric artery are identified, and one to three branches are divided depending on the required length (Fig. 1D). Prior to each division, the vessels are clamped, and indocyanine green (ICG; Verdye^®^, Diagnostic Green GmbH, Aschheim-Dornach, Germany) is injected intravenously to assess perfusion of the jejunal arcade (Fig. 1E). The right pleura is opened transhiatally under direct visualization, and the long Roux limb is introduced into the mediastinum, either in an ante- or retrocolic fashion. In order for it to not fall down or rotate during repositioning of the patient and to “know” how much you can pull it up in the thorax, it is secured to the right crus using a Vicryl^®^ suture (Ethicon, Johnson & Johnson MedTech, Somerville, NJ, USA) (Fig. 1F). A stapled enteroanastomosis is then constructed approximately 60–70 cm distally of the level of the jejunal alimentary transection. A closed-suction Jackson-Pratt^®^ drain (Cardinal Health, Dublin, OH, USA) is advanced to the mediastinum along the Roux limb. A feeding jejunal catheter is inserted. If the esophagus has already been transected in this phase, the specimen is extracted through a small Pfannenstiel incision, thereby completing the abdominal phase.

**Fig. 2:**
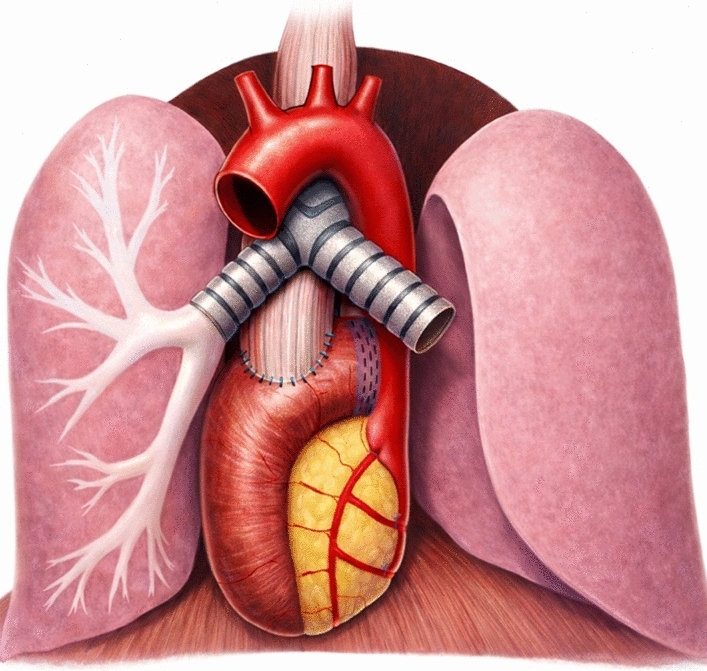
The final result with an end-to-side esophagojejunostomy high up in the mediastinum, here with two divided jejunal arteries. © Adam Zeyara, MD

#### Thoracic phase

The patient is repositioned to a semiprone “crawl” position, and single-lung ventilation is initiated. Robotic ports are placed (four 8 mm robotic ports, and if the specimen was not extracted during the abdominal phase, one of these should be a 12 mm port to allow for stapling), along with an 8 mm assistant port. See Fig. 1G for trocar positions. A paravertebral block catheter is inserted into the subpleural space for regional anesthesia. The esophageal mobilization is then completed, and a thoracic D2 lymphadenectomy is performed if indicated (paraesophageal and subcarinal stations). If not already divided, the esophagus is transected here using the SureForm™ robotic stapler (blue cartridge). An end-to-side hand-sewn esophagojejunostomy is created using two running 3–0 V-Loc™ barbed sutures (Medtronic, Minneapolis, MN, USA) and then reinforced with interrupted 4–0 PDS™ II monofilament sutures (Ethicon, Johnson & Johnson MedTech) (Fig. 1H). If the specimen has not been removed earlier, it is extracted through a small thoracotomy at the assistant port site using an Alexis® wound retractor (Applied Medical, Rancho Santa Margarita, CA, USA). The Jackson-Pratt^®^ drain (introduced during the abdominal phase) is now positioned along the anastomosis. A pleural flap is sutured over the anastomosis with a running 4.0 PDS™. A schematic illustration of the final reconstruction is shown in Fig. 2.

**Fig. 3:**
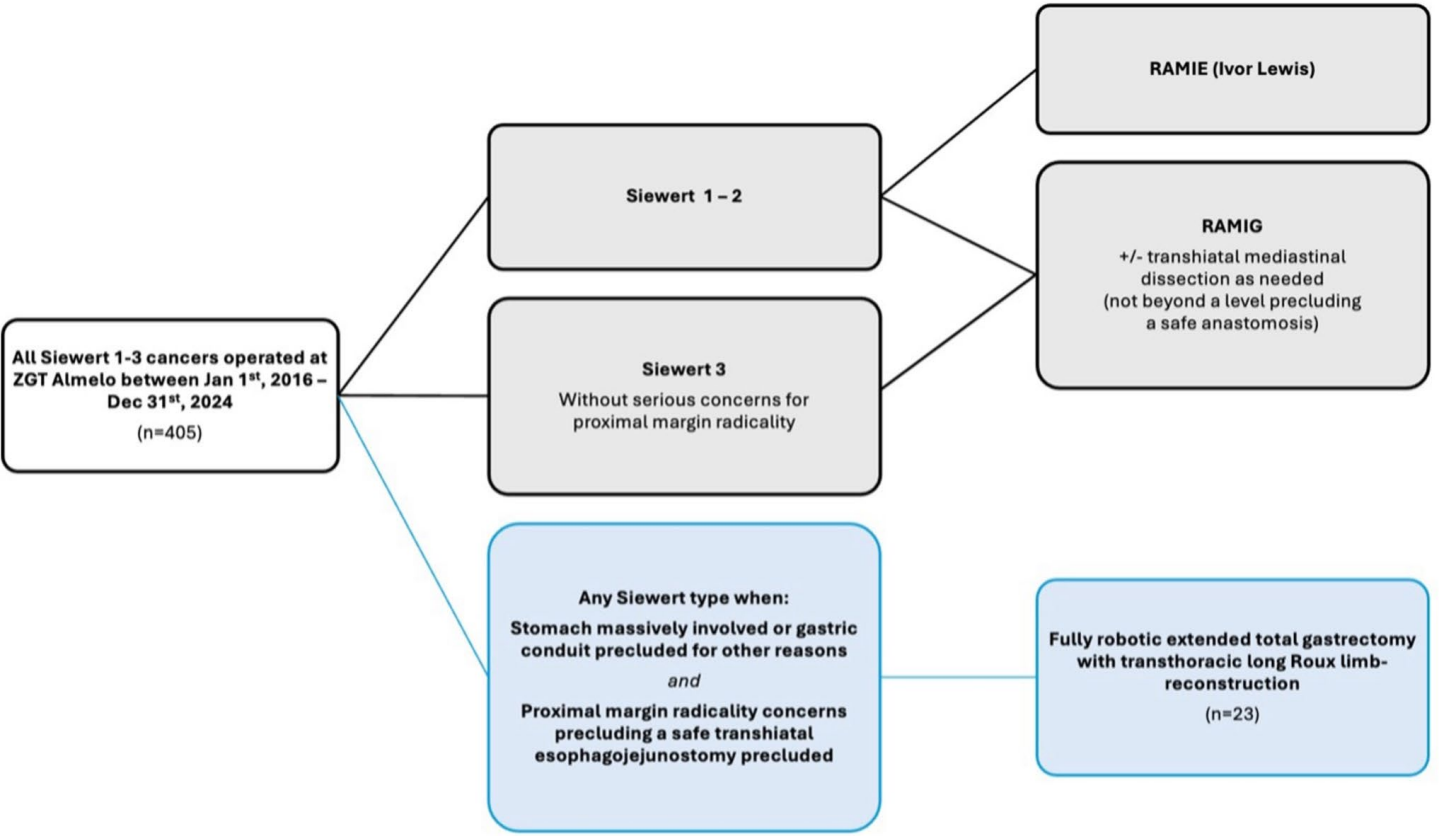
This algorithm/flowchart was made to contextualize the fully robotic extended total gastrectomy with transthoracic long Roux limb-reconstruction in the spectrum of treatment options of junctional cancers. As shown in the figure, the technique was solely employed for patients with junctional cancers where a gastric conduit was precluded (whatever the reason) and when there were proximal margin radicality concerns to such a degree that a safe abdominal/transhiatal esophagojejunostomy could not be performed. These patients would traditionally have been considered for open surgery with a long Roux-limb or a colonic interposition

## Results

Of all the Siewert 1–3 tumors (*n* = 405) that were treated with any resection during the study period (January 1st, 2016, to December 31st, 2024), a total of 23 patients were included in the analysis. To contextualize the presented technique in the spectrum of treatment options of junctional cancers, an algorithm/flowchart was drawn (Fig. 3). As shown in the figure, the technique was solely employed for patients with junctional cancers where a gastric conduit was precluded (whatever the reason) and when there were proximal margin radicality concerns to such a degree that a safe abdominal/transhiatal esophagojejunostomy could not be performed. These patients would traditionally have been considered for open surgery with a long Roux limb or a colonic interposition.

Most patients presented with locally advanced disease (cT3-4 *n* = 21, and *N* + *n* = 18) and exhibited unfavorable histological features (diffuse, mucinous or mixed type *n* = 12). Full patient data and clinical tumor characteristics are available in Table 1**.**

**Table 1:** Patient data and clinical tumor characteristics

Mean age, years (95% CI)	65.8 (59.8 – 71.9)
Gender, *n*	
M	15
F	8
ASA class, *n*	
1	1
2	11
3	11
Tumor epicenter, *n*	
Distal esophagus	5
Junctional	13
Stomach	5
cT, *n*	
T2	2
T3	19
T4	2
cN, *n*	
N0	5
N1	10
N2	7
N3	1
Histology, *n*	
Intestinal type adenocarcinoma	8
Diffuse type adenocarcinoma	10
Mucinous type adenocarcinoma	1
Mixed type adenocarcinoma	1
Adenocarcinoma, subtype unavailable	2
Squamous cell carcinoma	1
Neoadjuvant treatment, *n*	
CROSS	12
FLOT	10
Other	1

The mean operative time was seven hours. The median length of stay in the intensive care unit (ICU) and hospital was 1 day and 9 days, respectively. Full operative data are available in Table 2**.**

**Table 2:** Operative data

Mean operative time, minutes (95% CI)	424.3 (383.5 – 465.1)
Mean blood loss, ml (95% CI)	95.8 (64.7 – 126.9)
Jackson-Pratt	
Yes	23
Chest tube, *n*	
Yes	10
No	13
Mean length of Roux limb, cm (95% CI)	61.8 (57.3 – 66.3)
Frozen section, *n*	
Yes	21
No	2
D2 lymphadenectomy (abdominal), *n*	
Yes	23
Roux limb position, *n*	
Antecolic	5
Retrocolic	17
Intraoperative endoscopy, *n*	
Yes	1
No	22
Firefly (ICG) used for Roux limb, *n*	
Yes	21
No	2

Major postoperative complications, defined as Clavien–Dindo grade ≥ 3, occurred in 5 patients (22%). Anastomotic leakage occurred in 2 patients (8.7%). There was no 30-day mortality, and the 90-day mortality rate was 9% (*n* = 2). Both 90-day deaths were related to postoperative pneumonia, and neither was directly attributable to anastomotic failure. R0 was defined as no tumor at inked resection margins (proximal, distal as well as CRM). Despite negative intraoperative frozen sections, five R1 resections were identified on final pathological assessment. All of these tumors demonstrated a diffuse histological subtype. The mean lymph node yield was 26.5. Full postoperative clinical outcomes and pathology report data are available in Table 3**.**

**Table 3:** Postoperative clinical outcomes and pathology report data

Median postoperative ICU stay, days (range)	1 (0 – 7)
Median length of stay, days (range)	9 (3 – 37)
Clavien–Dindo, *n*	
0	12
1	1
2	5
3	2
4	3
Anastomotic leak, *n* (%)	2 (8.7)
Pneumonia, *n* (%)	5 (21.7)
R1, *n* (%):	5 (22) All proximal All had diffuse histology All had negative frozen sections
ypT, *n*	
ypT0	3
ypT1	0
ypT2	3
ypT3	13
ypT4	4
ypN, *n*	
ypN0	7
ypN1	5
ypN2	2
ypN3	9
Mean lymph node yield (abdominal), total, *n* (95% CI):	26.5 (23 – 30)
Mandard TRG grade, *n*	
1	3
2	4
3	11
4	2
5	3

**Fig. 4:**
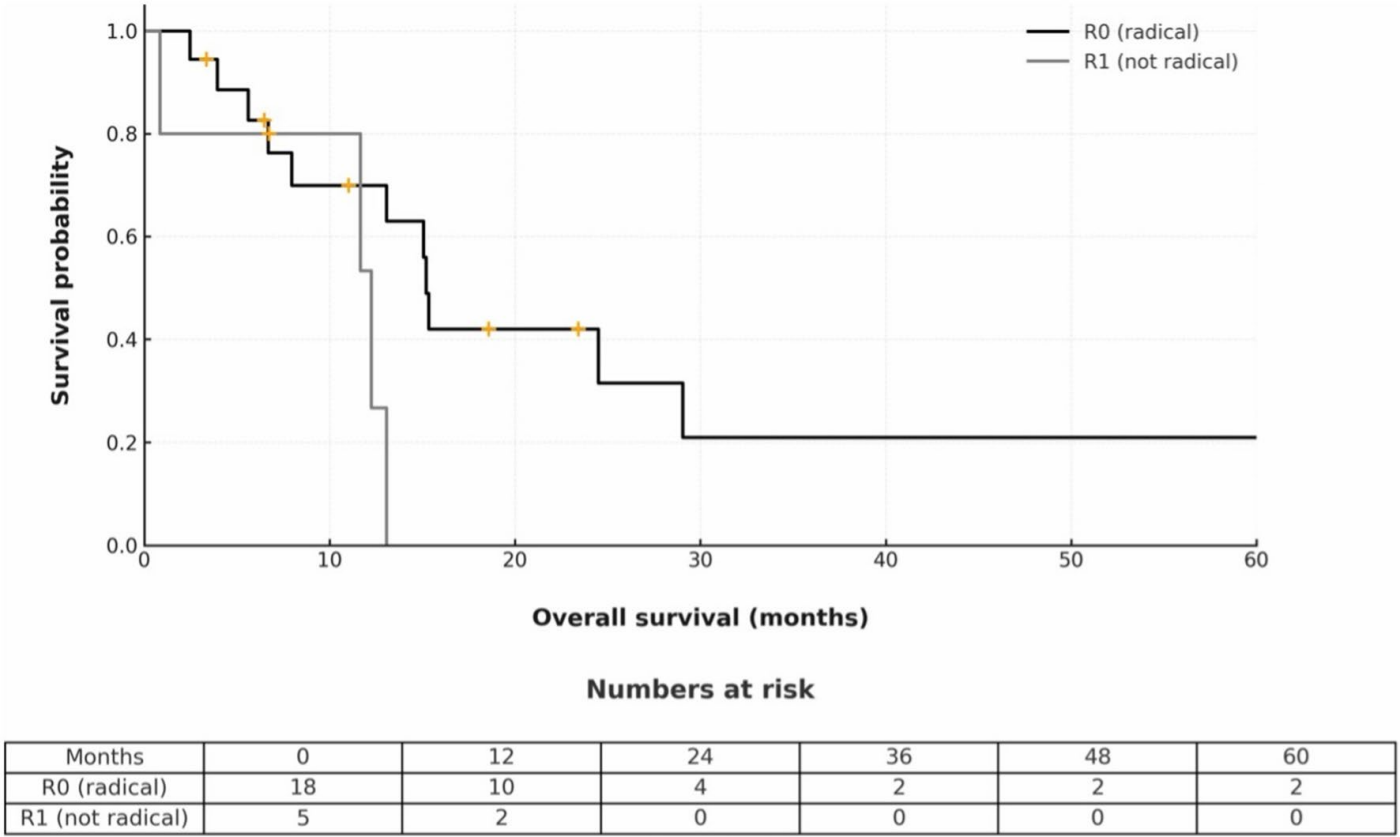
Kaplan–Meier curve of overall survival

**Fig. 5:**
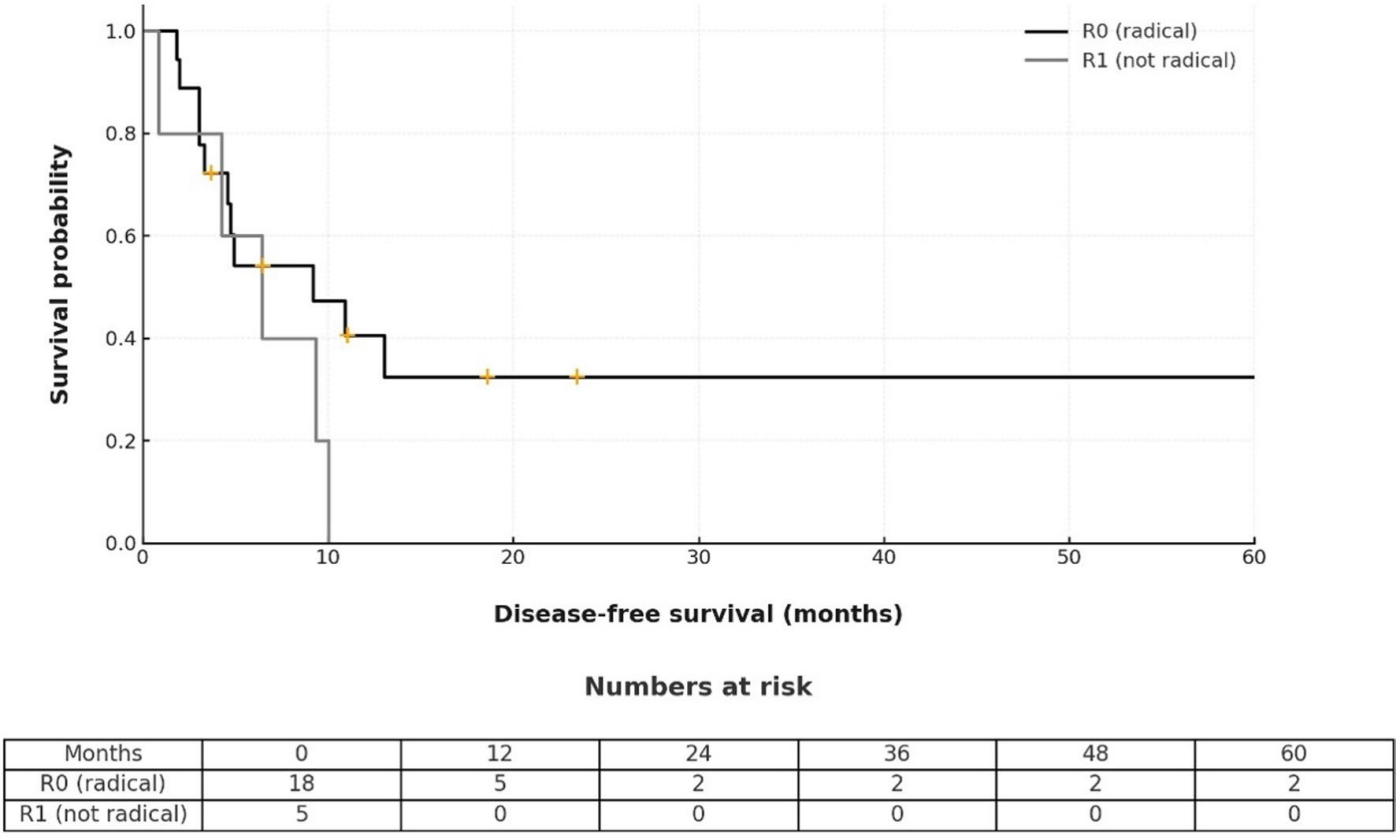
Kaplan–Meier curve of disease-free survival

16 patients (69.6%) suffered a recurrence within the first year and the most common site of recurrence was the peritoneum. The median overall survival (including R1 resection) was 13.1 months, and the median disease-free survival was 6.4 months, including the R1 resections. In R0 resections only, overall survival was 15.2 and disease-free survival 9.2 months. Table 4 and Figs. 4 and 5 show recurrence and survival data.

**Table 4:** Recurrence and survival data

Recurrence	
< 12 months, *n* (%)	16 (69.6)
> 12 months, *n*	6
Unavailable, *n*	1
Recurrence site	
Local	1
Single solid organ (lung, liver, spleen)	2
Peritoneal carcinomatosis	6
Pleural carcinomatosis	1
Multiple	5
Unavailable	8
30-day survival, *n* (%)	23 (100)
90-day survival, *n* (%)	21 (91)
Median OS^a^, months (95% CI)	13.1 (9.1 – 17)
Median OS (R0 only, *n* = 18), months (95% CI)	15.2 (12.4 – 18)
Median DFS^b^, months (95% CI)	6.4 (0.1 – 12.8)
Median DFS (R0 only, *n* = 18), months (95% CI)	9.2 (1.1 – 17.2)

^a^Overall survival, ^b^Disease-free survival

## Discussion

This series describes the first fully robotic technique for extended gastrectomy with a transthoracic long Roux limb as an alternative to open two-field or thoraco-phrenico-abdominal approaches. Given the technical complexity of the procedure, the intended audience is high-volume robotic esophagogastric surgeons. Accordingly, the present study does not advocate broad adoption but rather aims to describe a feasible and reproducible minimally invasive option for carefully selected patients.

Moreover, the oncologic and survival outcomes reported in this series should be interpreted with caution. The present cohort represents a highly selected subset of patients with predominantly locally advanced disease and unfavorable histological subtypes, most notably diffuse-type adenocarcinoma. In addition, the limited sample size precludes meaningful comparative or adjusted survival analyses. Consequently, survival outcomes are presented descriptively and primarily serve to contextualize the biological aggressiveness of the treated tumors rather than to evaluate oncologic superiority or equivalence of the surgical approach.

Outcomes indicate that the technique is feasible and safe with adequate short-term outcomes. The median ICU time was one day, and the median total length of stay was nine days, comparable to previous series on thoracolaparoscopic approach [1]. Recent literature on the open approach is limited; however, in a Swedish study of 83 patients from 2019, the median total length of stay was 16 days, suggesting that the minimal invasiveness of the technique may enhance postoperative recovery [15].

The two observed anastomotic leaks qualify as type C leaks, as their management required both endoscopic stent placement as well as thoracoscopic pleural debridement. The defects were confined and there was no evidence of serious ischemia or necrosis of the long Roux limbs. Neither of the two patients were deceased within 90 days after surgery.

As suggested by the relatively high number of R1 resections and the fact that more than two-thirds suffered a recurrence within 12 months, the studied sample was indeed a distillate of advanced cases (most cT3-4N + with diffuse histology). As expected, all R1 resections occurred at the proximal margin, all of which had a diffuse histology and a negative intraoperative frozen section as well as an intraoperative endoscopy to guide the appropriate level of resection. These findings highlight the ongoing challenge of achieving negative margins in diffuse tumors, even with thoracic extension and frozen section assessment and intraoperative endoscopy guidance.

Median overall and disease-free survival for the entire sample were 13.1 and 6.4 months, respectively. In R0 resections only (*n* = 18), they were 15.2 and 9.2, respectively. These outcomes are comparable to previously reported figures for advanced tumors with unfavorable histological subtypes [16]. Given the small sample size and the fact that most patients had cT3–4N + tumors with poor histology, survival analyses were not adjusted for tumor stage.

Despite being the first series on a fully robotic technique, it remains underpowered in several respects, suffering from typical weaknesses inherent to all small sample sizes such as inflated type I/II errors, lack of representativeness, and violations of statistical assumptions. Keeping this in mind, it should primarily be seen as a descriptive study of a novel surgical technique.

In summary, the fully robotic extended total gastrectomy with transthoracic long Roux limb reconstruction appears feasible and safe, combining the advantages of minimally invasiveness in recovery with the enhanced precision of robotic articulation. Despite these advantages as well as intraoperative endoscopic and pathological assessment, a relatively high rate of proximal R1 resections persisted—comparable to previously reported open series—reflecting the infiltrative biology of diffuse-type tumors rather than a limitation of the technique.

Ultimately, the approach achieved what it was designed to do a controlled, high thoracic exposure for a neat proximal dissection and a secure intrathoracic anastomosis, extending beyond the anatomic limits of a purely transhiatal strategy, aiming at reduced morbidity of open two-field surgery.

## Supplementary Information

Below is the link to the electronic supplementary material.

Supplementary file1 (MOV 344442 KB)

## Data Availability

All data are available upon reasonable request.

## Abbreviations

CI: Confidence interval
DFS: Disease-free survival
ICG: Indocyanine green
ICU: Intensive care unit
IQR: Interquartile range
OS: Overall survival
SD: Standard deviation
